# Regulation of extracellular matrix components by AmrZ is mediated by c-di-GMP in *Pseudomonas ogarae* F113

**DOI:** 10.1038/s41598-022-16162-x

**Published:** 2022-07-13

**Authors:** Esther Blanco-Romero, Daniel Garrido-Sanz, David Durán, Rafael Rivilla, Miguel Redondo-Nieto, Marta Martín

**Affiliations:** 1 Departamento de Biología, Facultad de Ciencias, Universidad Autónoma de Madrid, Darwin, 2, 28049 Madrid, Spain; 2 Department of Fundamental Microbiology, University of Lausanne, 1015 Lausanne, Switzerland

**Keywords:** Genetics, Microbiology, Molecular biology, Plant sciences

## Abstract

The AmrZ/FleQ hub has been identified as a central node in the regulation of environmental adaption in the plant growth-promoting rhizobacterium and model for rhizosphere colonization *Pseudomonas ogarae* F113. AmrZ is involved in the regulation of motility, biofilm formation, and bis-(3′-5′)-cyclic dimeric guanosine monophosphate (c-di-GMP) turnover, among others, in this bacterium. The mutants in *amrZ* have a pleiotropic phenotype with distinguishable colony morphology, reduced biofilm formation, increased motility, and are severely impaired in competitive rhizosphere colonization. Here, RNA-Seq and qRT-PCR gene expression analyses revealed that AmrZ regulates many genes related to the production of extracellular matrix (ECM) components at the transcriptional level. Furthermore, overproduction of c-di-GMP in an *amrZ* mutant, by ectopic production of the *Caulobacter crescentus* constitutive diguanylate cyclase PleD*, resulted in increased expression of many genes implicated in the synthesis of ECM components. The overproduction of c-di-GMP in the *amrZ* mutant also suppressed the biofilm formation and motility phenotypes, but not the defect in competitive rhizosphere colonization. These results indicate that although biofilm formation and motility are mainly regulated indirectly by AmrZ, through the modulation of c-di-GMP levels, the implication of AmrZ in rhizosphere competitive colonization occurs in a c-di-GMP-independent manner.

## Introduction

*Pseudomonas ogarae* F113, formerly known as *P. fluorescens* F113^1^, was isolated from the sugar-beet rhizosphere and is considered a plant-growth-promoting and biocontrol rhizobacterium^2^. This bacterium can colonize the roots of many plants and is used as a model for rhizosphere colonization^3–5^. The adaption of F113 to the rhizosphere environment is partially dependent on two master transcription factors (TFs), AmrZ and FleQ, and a second messenger molecule, bis-(3′-5′)-cyclic dimeric guanosine monophosphate (c-di-GMP)^6,7^. The transition between motile and sessile lifestyles is also controlled by the levels of this second messenger, which are in turn controlled by the activity of diguanylate cyclases (DGCs), containing GGDEF domains, involved in c-di-GMP synthesis and phosphodiesterases (PDEs), which contain EAL or HD-GYP domains and degrade c-di-GMP^8,9^. High c-di-GMP levels are associated with a sessile lifestyle and biofilm formation, whereas low levels of this second messenger are responsible for motility and a planktonic lifestyle^10^. The role of this molecule in modulating rhizosphere colonization and attachment to the plant has been demonstrated in F113, *Pseudomonas putida* KT2440, and *Pseudomonas syringae*^11–13^. AmrZ has been described as a regulator of more than half of the DGCs and PDEs encoded in the F113 genome and its mutant is deficient in c-di-GMP production^7^.

AmrZ belongs to the Arc superfamily of proteins and possesses a ribbon-helix-helix DNA binding domain, a flexible N-terminus, and a C-terminal domain^14^. AmrZ is ubiquitously found in *Pseudomonas* and its activity depends on the formation of tetramers mediated by its C-terminal domain^15^. The AmrZ protein was first described as a regulator of the production of the exopolysaccharide (EPS) alginate^14,16^. Later, it has been shown to regulate several other functions, including motility and c-di-GMP metabolism in different pseudomonads^7,15,17–21^ functioning either as an activator or a repressor of the gene expression^22^ and with opposing roles in different strains^23^. In F113, AmrZ is involved in the regulation of different functions such as motility, biofilm formation, c-di-GMP production, or iron homeostasis^7,24^. Conversely, this protein has been associated with repression of c-di-GMP synthesis in *P. aeruginosa*^19,20^ and *P. syringae* pv. *tomato* DC3000, being the former a negative transcriptional regulator of cellulose synthesis and an activator of alginate production, motility, and virulence^21^. In *P. aeruginosa* and F113, AmrZ has been identified as a negative transcriptional regulator of FleQ, the master regulator for flagellar synthesis^18,20,25,26^. FleQ is another bifunctional TF, able to act as a transcriptional activator or repressor^27^. This protein is recognized as a c-di-GMP effector, able to bind c-di-GMP^28^. The TF FleQ can inversely regulate swimming motility and EPSs production in a c-di-GMP-dependent manner in several species of pseudomonads^27,29–31^. In F113, AmrZ and FleQ constitute a regulatory hub in which they mutually and transcriptionally repress each other while conversely regulating other functions: AmrZ down-regulates motility and up-regulates putative extracellular matrix (ECM) components production whereas FleQ up-regulates motility and down-regulates putative ECM components production^6^. A similar antagonistic role of both TFs has also been shown by RNA-Seq during rhizosphere colonization^32^.

The ECM is the structure that binds cells in biofilms, acting mainly as a scaffold of these three-dimensional structures. It is composed mostly of different EPSs, extracellular proteins, and DNA^33^. The ECM of F113 is predicted to contain alginate, levan, *Pseudomonas* acidic polysaccharide (Pap), and poly-β-1, 6-N-acetylglucosamine (PNAG) polysaccharides, functional amyloids in *Pseudomonas* (Fap), large adhesion protein A (LapA) and medium adhesion protein A (MapA) adhesins, the putative extracellular mannuronan C-5 epimerase PsmE, and the fimbrial low-molecular-weight protein/tight adherence (Flp/Tad) pili^34^. Since the *amrZ* mutant is defective in biofilm formation^7^ and AmrZ has been found as a regulator of some putative ECM components in liquid cultures and rhizosphere^7,32^, this work aimed to investigate the role of AmrZ in the regulation of the ECM components in F113. Additionally, we have analyzed the implication of c-di-GMP in this AmrZ-mediated regulation and the impact of this second messenger in the AmrZ-associated phenotypes of biofilm formation, motility, and rhizosphere colonization in F113. Finally, we have also studied the cross-regulation of AmrZ and FleQ in the regulation of ECM components and biofilm-related phenotypes.

## Results

### AmrZ regulates the expression of genes encoding ECM components in *P. ogarae* F113

**Figure 1:**
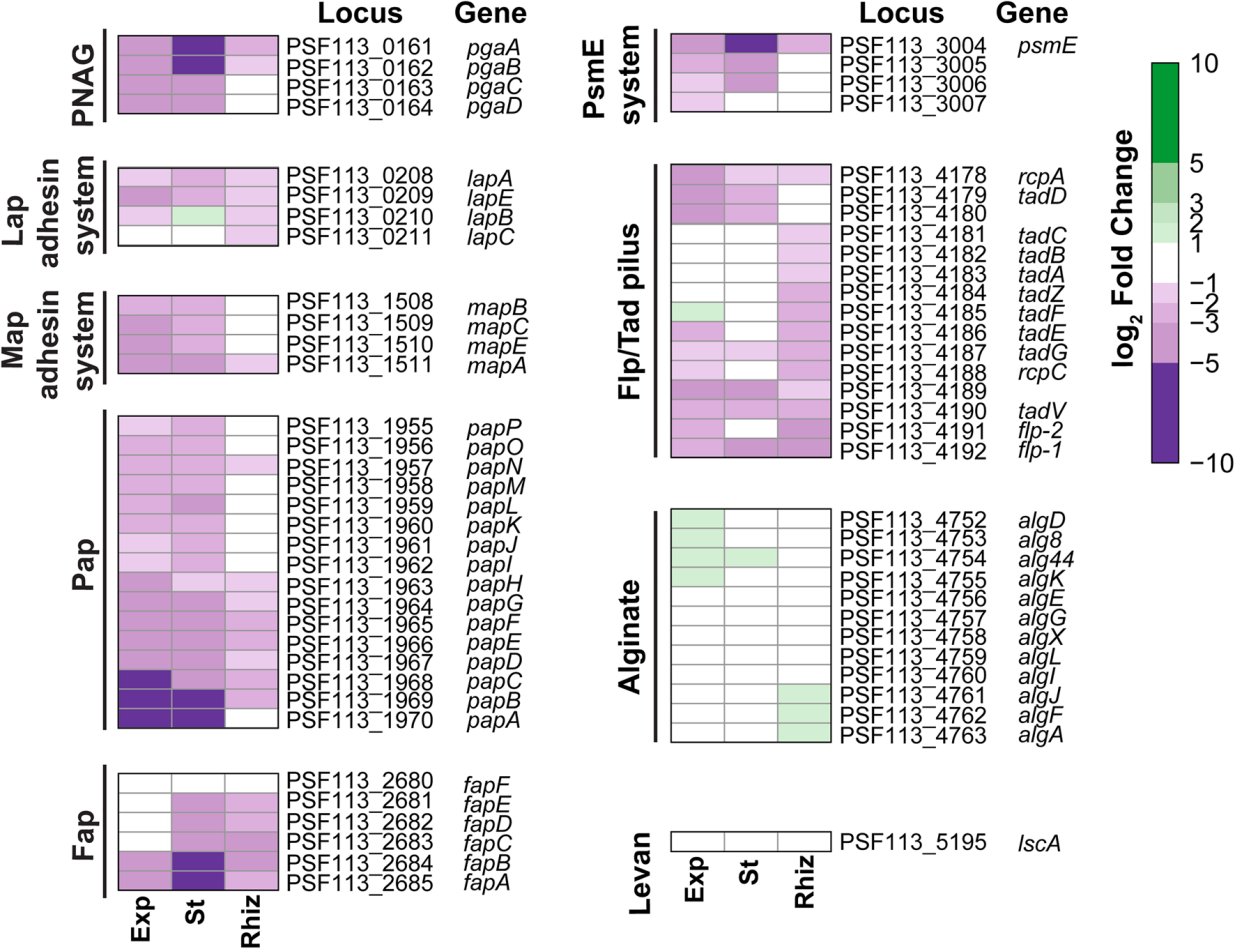
AmrZ is a transcriptional regulator of ECM components in *P. ogarae* F113. Heatmap displaying the differential gene expression found in the *amrZ* mutant taking *P. ogarae* F113 wild-type as the reference at different growth conditions: exponential (Exp) or stationary (St) liquid cultures or rhizosphere (Rhiz). The genes included in this analysis putatively encode ECM components^34^. The heatmap includes the log_2_ Fold-Change (*amrZ*^*-*^/wild type) values obtained in RNA-Seq analysis and are included in Supplementary Table S1. Color scale: green, negative regulation by AmrZ; white, no regulation; purple, positive regulation by AmrZ.

As previously observed, *amrZ* mutants are dramatically impaired in the attachment to surfaces and present a different morphology compared to the wild-type strain in the presence of Congo Red (CR)^7^. Since these observations are consistent with a defect in the production of ECM components, we have studied the transcriptomic profile of F113 and its isogenic *amrZ* mutant using RNA-Seq approach^32^. The RNA-Seq analyses were done under different growth conditions: exponential and stationary phases in liquid cultures and after rhizosphere colonization. As observed in Fig. 1, the transcriptomic analyses have shown that the expression of several gene clusters encoding proteins implicated in polysaccharide synthesis and the production of extracellular structural proteins is downregulated in the *amrZ* mutant under all tested conditions compared to the wild-type strain. Differential gene expression was found for 59 genes putatively involved in the production of eight different ECM components, mostly observed during stationary phase, indicating a role for AmrZ in activating the expression of genes related to ECM during this phase (Fig. 1). Fold-Change and p-adjusted values for those genes are shown in Supplementary Table S1.

The AmrZ-regulated clusters include *papA-P* (PSF113_1970-1955) involved in the production of the putative Pap polysaccharide and *pgaA-D* (PSF113_0161-0164) encoding PNAG EPS. Some of the genes from the *alg* cluster (PSF113_4752-4763), involved in alginate synthesis, were differentially expressed in the *amrZ* mutant compared to the wild-type strain with log_2_ Fold-Change values close to the threshold under the tested conditions. The other F113-encoded polysaccharide, levan, is not subjected to AmrZ regulation under any of the tested conditions. Regarding the regulated-clusters related with the production of extracellular proteins or proteinaceous structures, they include *fapA-F* (PSF113_2680-2685), which are necessary to produce Fap; PSF113_0208, PSF113_1511 and PSF113_3004 encoding the extracellular proteins LapA, MapA, and PsmE, respectively; as well as their associated transport systems (PSF113_0209-0211, PSF113_1508-1510, and PSF113_3005-3007); and PSF113_4178-4192 involved in the Flp/Tad pilus formation.

### c-di-GMP governs the AmrZ-dependent regulation of ECM components in *P. ogarae* F113

We have previously shown that AmrZ is a major regulator of c-di-GMP synthesis and that AmrZ-mediated transcriptional activation of several DGCs occurs mainly during stationary phase^7^. On the other hand, almost all the ECM-related clusters have a FleQ-binding site^6^ in their promoter regions and only some of them harbor an AmrZ-binding site^24^. Moreover, we have previously described the reciprocal regulation between AmrZ and FleQ TFs^6^. For these reasons, we hypothesized that the observed AmrZ regulation of EMCs could be done through the regulation of c-di-GMP levels or its interrelation with FleQ. In order to test this hypothesis, we first artificially increased the overall c-di-GMP concentration in the wild-type strain, the *amrZ* mutant, and a double mutant in *amrZ* and *fleQ*. The increase of c-di-GMP concentration was obtained through ectopic expression of the mutated version of the pleD* gene from *Caulobacter crescentus* encoding a constitutive DGC^35,36^. It has been shown that pleD* overexpression stimulates EPSs production, such as cellulose in *P. syringae* pv. *tomato* DC3000^13^.

**Figure 2:**
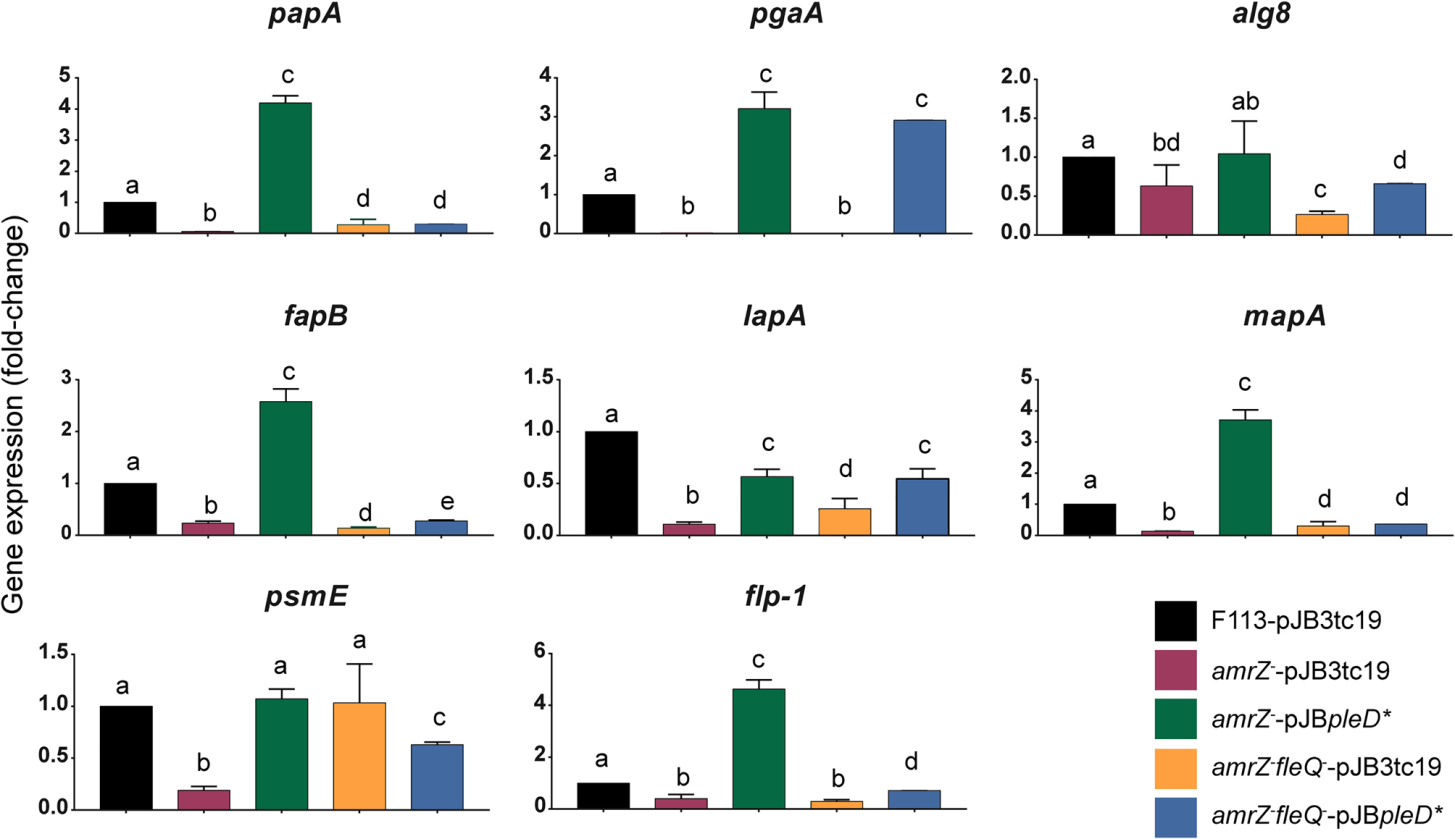
AmrZ regulates ECM-related genes in a c-di-GMP- and FleQ-dependent manner. qRT-PCR analysis of selected extracellular matrix-related genes in stationary phase. Gene expression from *amrZ*^-^ or *amrZ*^-^*fleQ*^-^ carrying pJB3Tc19 or pJBpleD* was normalized to *rpoZ* and relativized to the wild-type strain carrying the empty plasmid pJB3Tc19. Averages and standard deviation (SD) from two biological replicates with three technical replicas are represented. Means not sharing any letter are significantly different by multiple t-tests with Bonferroni-Dunn method (*p* value < 0.05).

We have also used a qRT-PCR approach to test the expression of selected genes encoding putative ECM components in the wild-type strain and derivatives growing in SA medium at stationary phase either in the absence or presence of pleD* (Fig. 2). As shown in Fig. 2, gene expression was remarkably low, and in some cases, almost undetectable for most of the ECM-related genes tested in *amrZ*^*-*^ and *amrZ*^*-*^*fleQ*^*-*^ carrying the empty plasmid pJB3Tc19, validating RNA-Seq results. In the case of *psmE* (PSF113_3004), its gene expression is much higher in the double mutant than in the single mutant, suggesting that FleQ is repressing its expression. Conversely, the presence of pleD* in the *amrZ* mutant resulted in a significant increase in the expression levels of all tested genes. This increase in expression was several folds higher than in the wild-type for genes encoding Pap (*papA*, PSF113_1970), PNAG (*pgaA*; PSF113_0164), Fap (*fapB*; PSF113_2684), MapA (*mapA*, PSF113_1511), and Flp/Tad (*flp-1,* PSF113_4192), demonstrating that the role of AmrZ in the regulation of ECM components occurs indirectly through c-di-GMP. Interestingly, the expression of *lapA* (PSF113_0208), which encodes the large adhesin LapA was higher in the *amrZ*^*–*^pJBpleD* background than in the *amrZ* mutant, although expression was still lower than in the wild-type strain, suggesting additional elements involved in its regulation. However, the artificial increase in c-di-GMP levels was not enough to increase gene expression levels in the *amrZ*^-^*fleQ*^-^ background in *papA*, *alg8*, *fapB*, *lapA*, *mapA*, and *flp-1*, also suggesting the dependency of FleQ in their regulation. These results confirm that AmrZ mainly regulates ECM production indirectly, through the increase of c-di-GMP levels and with the interplay of the TF FleQ.

**Figure 3:**
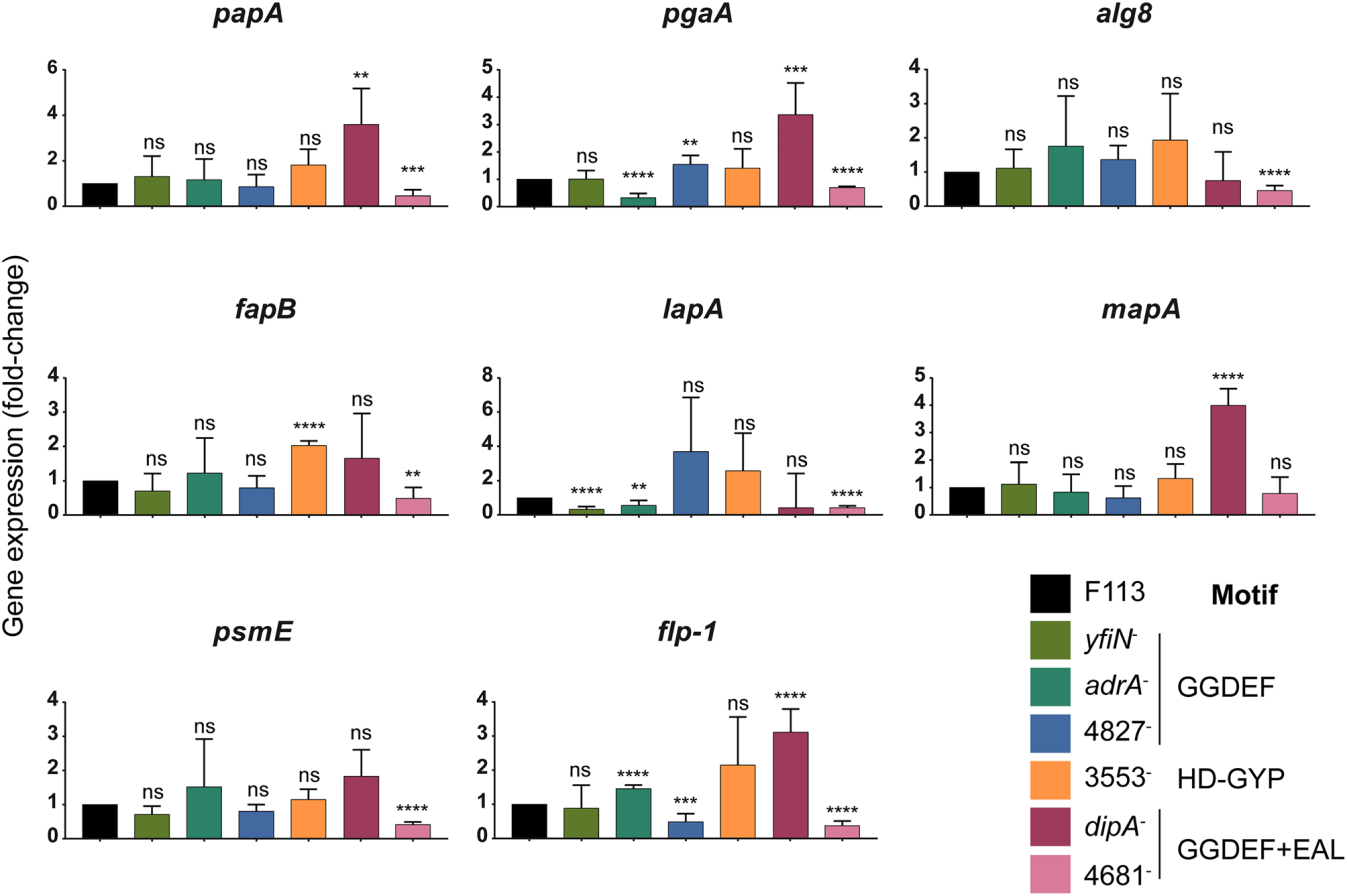
Local DGCs and PDEs are involved in the regulation of ECM-related genes. qRT-PCR analysis of selected extracellular matrix-related genes in stationary phase. Gene expression from mutants affected in the synthesis of proteins with GGDEF motifs (*yfiN*^-^, *adrA*^-^, and PSF113_4827), HD-GYP domain (PSF113_3553), or containing both GGDEF and EAL motifs (*dipA*^-^ and PSF113_4681) was normalized to *rpoZ* and relativized to the wild-type strain. Averages and SD from two biological replicates with three technical replicas are represented. Significant differences in gene expression were determined with multiple t-tests with Bonferroni-Dunn method (*p value < 0.05; **p value < 0.01; ***p value < 0.001; ****p value < 0.0001; ns: not significant).

The second approach used in this work aims to decipher which DGCs and PDEs are involved in the transcriptional regulation of ECM components in F113. For this purpose, we used mutants affected in the production of DGCs and PDEs with defects in biofilm formation and that have been previously reported as AmrZ-regulated targets: *dipA*^-^ (PSF113_0499), *yfiN*^-^ (PSF113_0715), *adrA*^-^ (PSF113_1982), PSF113_4827, PSF113_3553, and PSF113_4681^7^. DipA and PSF113_4681 contain both GGDEF and EAL motifs, YfiN, AdrA, and PSF113_4827 contain GGDEF motifs, and PSF113_3553 contains an HD-GYP domain. AmrZ activated expression of all the genes encoding these enzymes except for the *dipA* gene, for which a negative regulation was observed^7^. As shown in Fig. 3, the gene expression of ECM-related genes is also affected by changes in the pool of c-di-GMP produced by the DGCs and PDEs studied. Our results show that gene expression for *papA, pgaA* and *alg8* are decreased in PSF113_4681 mutant. Besides, *papA* and *pgaA* gene expression is increased in the *dipA* mutant; *pgaA* gene expression is increased in PSF113_4827 mutant and reduced in *adrA* mutant. Regarding protein components, *psmE, fapB, lapA* and *flp-1* gene expression is decreased in the PSF113_4681 mutant. Moreover, *fapB* gene expression is increased in the mutant PSF113_3553; *lapA* gene expression is reduced in *yfiN* and *adrA* mutants*; flp-1* gene expression is increased in *dipA* and *adrA* mutants and reduced in the PSF113_4827 and finally, *mapA* gene expression is exclusively increased in the *dipA* mutant (Fig. 3). These results show that different DGCs and PDEs participate in the regulation of the production of ECM components, by affecting the expression of genes involved in the synthesis of the ECM.

### Ectopic expression of pleD* restores the c-di-GMP related phenotypes in the *amrZ* and *amrZ*-*fleQ* mutants

We have previously reported that *amrZ* mutants show alterations in CR colony morphology and staining, are hypermotile, and are defective in the attachment to surfaces^7^. All these phenotypes are consistent with the low levels of c-di-GMP. To test whether these phenotypes were caused by the low levels of c-di-GMP that are prevalent in the *amrZ* mutant^7^, we have used ectopic expression of pleD* from a plasmid in the wild-type and *amrZ* mutant backgrounds. Furthermore, since FleQ is a regulator of motility and biofilm-related genes in this bacterium^6^ and there is a cross-regulation by AmrZ and FleQ of ECM-related genes expression according to the findings of this work, we have also tested the suppression by c-di-GMP of the CR, biofilm and motility phenotypes in the double mutant *amrZ-fleQ*.

**Figure 4:**
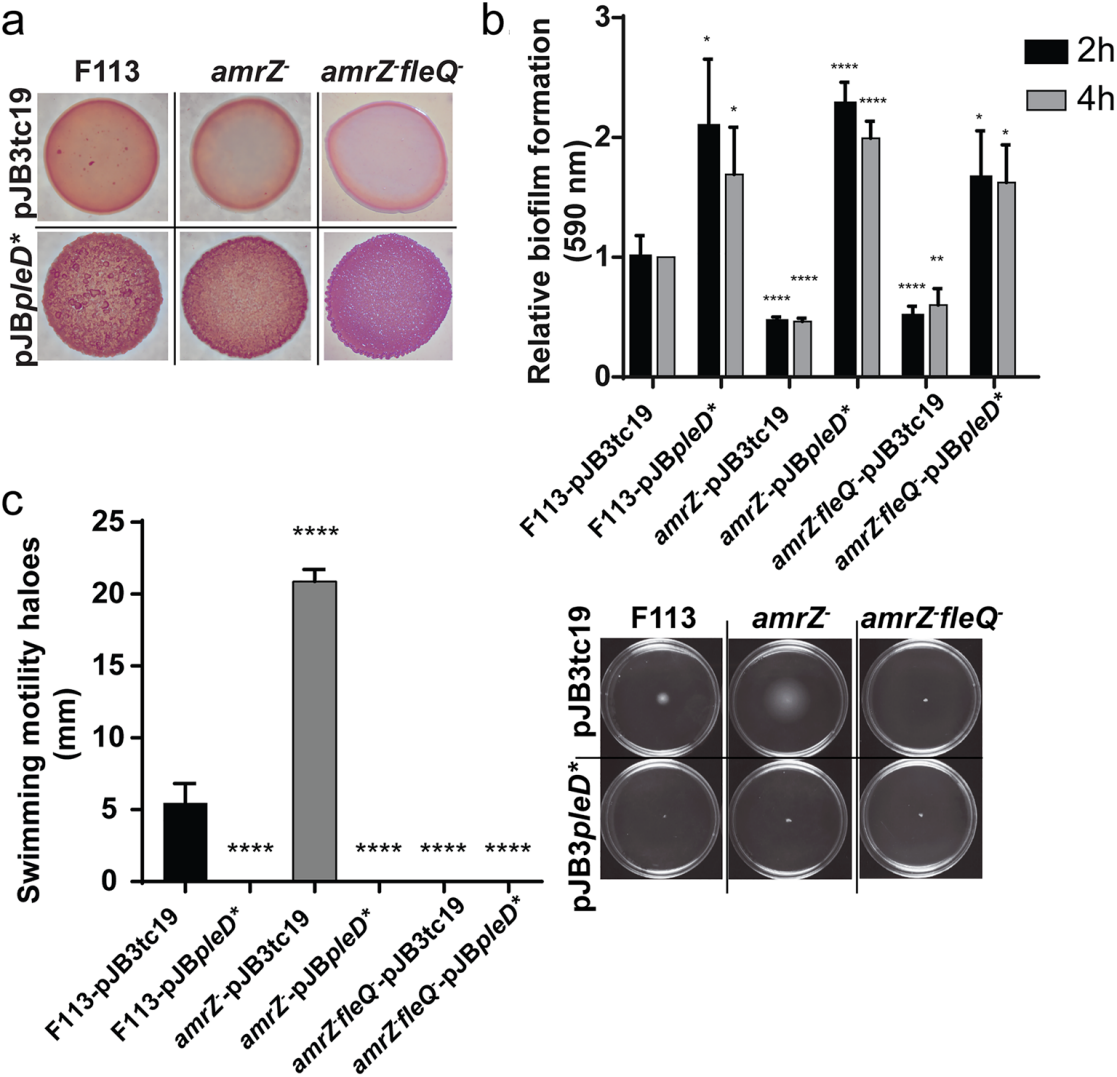
c-di-GMP restores the biofilm and motility phenotypes in *amrZ*^-^ and *amrZ*^-^*fleQ*^-^. (**a**) Morphology and Congo Red (CR)-binding pattern of *Pseudomonas ogarae* F113 and derivatives colonies carrying either the empty plasmid pJB3Tc19 or pJBpleD* grown for 48 h in Yeast-Mannitol-Broth (YMB) medium containing CR and Tc (zoom 1.25x). (**b**) Relative crystal violet-based biofilm formation assay of *P. ogarae* F113 and derivatives carrying pJB3Tc19 or pJBpleD* after 2 h and 4 h growth in microtiter plates with LB medium. The average and SD of four biological replicates with 16 technical replicates are represented; data was relativized to F113pJBTc19. (**c**) Swimming motility assay of *P. ogarae* F113 and derivatives carrying pJB3Tc19 or pJBpleD* at 24 h in SA medium containing Tc. Average and SD of three biological replicates with three technical replicates are shown. Images show swimming haloes formed after 24 h. Asterisks denote statistical significance of the data according to t-tests with Bonferroni-Dunn correction: *p value < 0.05; **p value < 0.01; ****p value < 0.0001.

As shown in Fig. 4, the phenotypes associated with *amrZ*^*-*^ and *amrZ*^-^*fleQ*^-^ are substantially modified when increasing intracellular levels of c-di-GMP by overexpression of pleD*. We analyzed the colony morphology and CR-binding ability of *amrZ*^*-*^ and *amrZ*^*-*^*fleQ*^*-*^ on CR-supplemented plates in the absence or presence of pleD* (pJB3Tc19 and pJBpleD* respectively). As shown in Fig. 4a, *amrZ*^*-*^ and *amrZ*^*-*^*fleQ*^*-*^, showed a light staining, a smooth patch, and an entire border. Conversely, the wild-type strain presented stronger staining, the patch had a rough texture, and its border was dented. In the wild-type, the overproduction of PleD* resulted in a rougher texture and slightly stronger staining. A similar phenotype was observed for all the mutants overexpressing pleD*: an increase in the color intensity, rougher texture, and dented borders. Furthermore, Fig. 4b shows that the defective biofilm formation phenotypes of the *amrZ*^*-*^ and *amrZ*^*-*^*fleQ*^*-*^ are reversed by increasing c-di-GMP intracellular levels, both after the initial attachment at 2 h and in a later development stage at 4 h. Indeed, not only attachment to solid surfaces was increased, but also cell–cell aggregates and pellicle formation (Supplementary Figures S1 and S2) were higher in the pleD* overexpressing strains. Finally, swimming motility analysis has shown the suppression of the hypermotile phenotype of the *amrZ* mutant caused by pleD* overexpression (Fig. 4c). Moreover, both F113-pJBpleD* and *amrZ*^*–*^pJBpleD* were non-motile after 24 h. As expected, *amrZ*^*-*^*fleQ*^*-*^ is non-motile in the absence or presence of pleD*, due to the crucial role of FleQ in the synthesis of the flagellar apparatus in this bacterium^4,6^. Altogether, these results suggest that AmrZ regulates motility and biofilm formation mainly through the regulation of c-di-GMP levels.

### Overproduction of c-di-GMP does not suppress the defect in rhizosphere competitive colonization in an *amrZ* mutant

**Figure 5:**
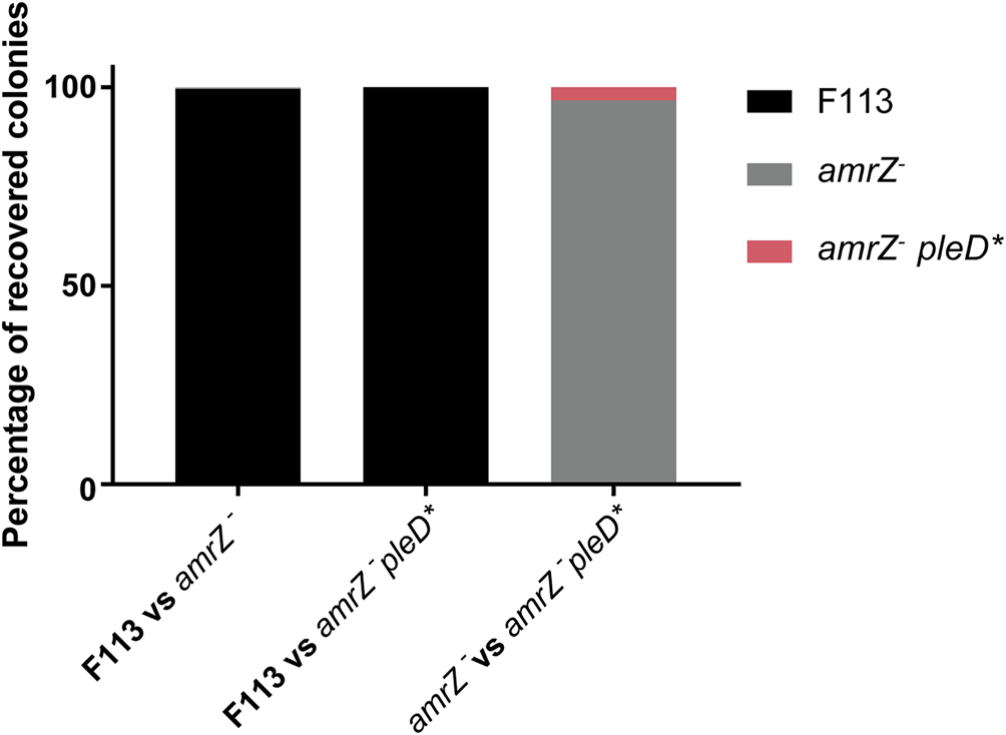
c-di-GMP does not restore the defective rhizosphere competitive colonization phenotype of an *amrZ* mutant. *Pseudomonas ogarae* F113 wild-type, *amrZ*^-^, and the *amrZ* mutant containing a miniTn7-based pleD* integration were tested in competitive colonization assays. CFUs recovered from the alfalfa rhizosphere after seven days post-inoculation were tested for antibiotic resistance to distinguish the different strains. Percentages of colonies are represented. Each experiment was repeated twice with ten plants per experiment.

Increased motility in *P. ogarae* F113 has been associated with enhanced rhizosphere competitive colonization ability^11^. However, despite *amrZ*^*-*^ being a hypermotile mutant, it is dramatically impaired in competitive rhizosphere colonization assays^7^. Thus, a similar approach as previously described in this work was carried out to understand the role of c-di-GMP in the AmrZ regulation of the rhizosphere competitive colonization process. To avoid plasmid loss due to the lack of selection, alfalfa rhizosphere competitive colonization assays were performed using F113, the *amrZ* mutant, and the *amrZ* mutant harboring a mini-*Tn7* based pleD* overexpression system (*amrZ*^*-*^ pleD*). The overexpression system was tested for the expected swimming and biofilm formation phenotypes (Supplementary Figure S3). As shown in Fig. 5, *amrZ*^*-*^ was totally displaced from the alfalfa rhizosphere by F113, consistent with our previous study^7^. Similarly, *amrZ*^*-*^ pleD* was displaced entirely by F113, indicating that an increase in c-di-GMP levels was not enough to suppress the competitive colonization phenotype of the *amrZ* mutant. Furthermore, *amrZ*^*-*^ pleD* is dramatically displaced even by the colonization-impaired *amrZ* mutant, suggesting that bacteria must tightly regulate c-di-GMP levels to be competitive during rizhosphere colonization.

## Discussion

Bacterial regulation of the switch between a planktonic lifestyle towards biofilm formation is an important trait for rhizosphere competence and persistence and, therefore, for the performance of rhizobacteria as plant growth promoters. When bacteria form biofilms, they are embedded in a self-produced ECM. The ECM components are subjected to fine control as they are needed not only in adhesion and immobilization of cells providing structure to biofilms but also play an essential role in signaling, genetic exchange, ion-sequestration, and migration^37^.

The second messenger c-di-GMP has been shown to play a crucial role in the transition from motile to sessile lifestyles^10^. We have previously shown that two pleiotropic regulatory proteins, AmrZ and FleQ, are involved in this transition in F113^6^. The involvement of c-di-GMP in this pathway is also evidenced by the regulation of DGC-coding gene expression exerted by AmrZ in this bacterium^7^, while FleQ is a c-di-GMP binding protein, whose activity depends on the binding of c-di-GMP as demonstrated in other pseudomonads^38^.

Here we have shown that in F113, AmrZ is a positive transcriptional regulator of genes related to ECM production, such as polysaccharides, Fap, extracellular adhesins, and the Flp/Tad pilus (Figs. 1 and 2) and that an increase in c-di-GMP levels in the *amrZ* mutant is enough to increase the expression to wild-type levels or higher (Fig. 2). Interestingly, only two of these genes, *lapA* and *flp-1* were previously found as putative direct regulatory targets for AmrZ due to the presence of an AmrZ-binding site in their promoter region^24^. In fact, in the case of *lapA*, c-di-GMP levels do not affect at the transcription level, suggesting a direct regulation by AmrZ. Four of the putative ECM components, Pap, Fap, MapA and Flp/Tad pili show a common regulatory pattern. AmrZ appears to regulate all of them via c-di-GMP production. Interestingly, in all of them c-di-GMP effect is FleQ dependent, since PleD* overproduction had no effect in their transcription in the *amrZ-fleQ* double mutant (Fig. 2). It is interesting to note that we have previously identified FleQ-binding sites in the promoter regions of *papA* (PSF113_1970),), *lapA* (PSF113_0208), *mapA* (PSF113_1511) *tadB* (PSF113_4182), and *rcpA* (PSF113_4178)^6^. These results indicate complex AmrZ-FleQ-c-di-GMP interplay participating in the regulation of these ECM components. AmrZ and FleQ have already been described as regulators of the synthesis of different ECM components in other pseudomonads^19,31,38–40^. A role for FleQ has been also observed for *lapA* expression in *P. putida* KT2440^6^. Conversely, we have not observed a role for FleQ in *psmE* transcription and the possible role of c-di-GMP on the regulation of *pgaA* appears independent of FleQ. Furthermore, in this work, we have shown that the expression of genes related to the production of ECM components responds to changes in the pool of c-di-GMP caused by mutation of distinct DGCs and PDEs that are regulated by AmrZ^7^ (Fig. 3). Genes *dipA* and PSF113_4681, encoding enzymes with both GGDEF and EAL domains appear to be the most important regulators for ECM components production. Mutants affected in either of these genes show an effect on the transcription of *papA*, *pgaA* and *flp1*, the *dipA* mutation affects *mapA* expression and the PSF113_4681 mutation has an effect on the transcription of *alg8, lapA* and *psmE*. In all cases, mutations in *dipA* resulted in an increased transcription, while mutations in PSF113_4681, resulted in a transcriptional decrease. Mutations affecting other genes encoding proteins with GGDEF domains (*adrA, yfiN* and PSF113_4827) and a HD-GYP domain (PSF113_3533) also affected the expression of several genes implicated in ECM components biosynthesis. In E*. coli*, PNAG production is also subjected to c-di-GMP-dependent transcriptional regulation via the c-di-GMP produced by the DGC YddV^41^. Similarly, c-di-GMP contribution to the alginate gene cluster expression has been previously observed in *E. coli*, in which the ectopic overexpression of the DGC YedQ led to increased *alg8* and *alg44* gene expression^42^. Although, to our knowledge, no previous data for the relation of c-di-GMP with the *fap* cluster in pseudomonads is available, regulation of their functional homologs in *Salmonella* sp. and *E. coli*, the amyloid curli fibers, is c-di-GMP dependent through the CsgD regulator^43,44^.

The results presented here have shown that modulation of c-di-GMP levels is the major pathway for the AmrZ regulation of biofilm formation and motility. In this regard, pleD* overexpression, leading to c-di-GMP overproduction, can suppress the biofilm-forming defects in the *amrZ* mutant, restoring attachment, Congo Red staining, and bacterial aggregation while suppressing the hypermotile phenotype (Fig. 4). However, the increase of c-di-GMP levels did not restore the rhizosphere colonization defect of the *amrZ* mutant (Fig. 5). We have previously shown that an *amrZ* mutant is totally displaced by the wild-type strain in competitive rhizosphere colonization experiments^7^. Here we show that the *amrZ*^-^ strain overproducing c-di-GMP is also displaced from the rhizosphere by the wild-type strain and by the colonization-defective *amrZ* mutant. These results clearly show that rhizosphere competitive colonization phenotype of the *amrZ* mutant is not a consequence of c-di-GMP levels, in contrast to biofilm formation and motility in the *amrZ* mutant, and that high levels of c-di-GMP seem to be detrimental for competitive rhizosphere colonization.

## Methods

### Bacterial strains and growth conditions

Bacterial strains and plasmids used in this work are listed in Supplementary Table S2. Overnight cultures of *Escherichia coli* were routinely grown in Luria Bertani (LB) medium^45^ at 37 °C. *Pseudomonas ogarae* F113 and derivatives were grown in LB, Sucrose-Asparagine (SA)^46^, or Yeast-Mannitol Broth (YMB) pH 6.8^47^ media, as indicated in each experiment, overnight at 28 °C with shaking. Purified agar (1.5% (w/v) for routine growth and Congo Red (CR) experiments or 0.3% (w/v) for swimming assays) or 1.5% (w/v) American Bacteriological Agar were used for solid medium. Antibiotics and dyes were added as required, at the following final concentrations: Rifampicin (Rif), 100 µg/mL, Cycloheximide (Chx), 10 μg/mL, Tetracycline (Tc), 70 μg/mL or 10 μg/mL for F113 or *E. coli*, respectively; Kanamycin (Km), 50 μg/mL for F113; Gentamicin (Gm), 10 μg/mL for *E. coli* or 5 μg/mL for F113; Chloramphenicol (Cm), 30 μg/mL and Ampicillin (Amp), 100 μg/mL for *E. coli*; and Congo Red (CR), 100 μg/mL.

Plasmid mobilization into F113 and derivatives was performed by triparental mating or electroporation. pJB3Tc19^48^ and pJBpleD*^13^ plasmids were introduced into F113 by triparental mating using *E. coli* JM109 as donor strain and using pRK600^49^. Similarly, miniTn7pleD*Tc^50^ was introduced in F113 by four-parental mating using pRK600 and pUX-BF13^51^.

### Swimming motility

Motility of *P. ogarae* F113 and derivatives carrying either the empty vector pJB3Tc19 or the overproducing diguanylate cyclase PleD*: pJBpleD* or miniTn7pleD*Tc was tested in swimming motility assays. Strains were grown overnight in SA agar plates supplemented with Tc at 28 °C. Strains were inoculated in the center of SA agar plates (0.3%) supplemented with Tc. Swimming haloes diameters were measured after 24 h incubation at 28 °C. Experiments were performed at least in duplicate with three replicates in each experiment.

### Congo red binding assay

pJB3Tc19 and pJBpleD* or miniTn7pleD*Tc-containing strains were grown overnight in YMB supplemented with Tc and 10 µL of culture were inoculated in triplicate in YMB agar plates supplemented with CR and Tc and incubated at 28 °C. Colony morphology and staining were recorded from day two to seven with a Leica MX125 stereoscope (zoom 1.25x). Every assay was performed three times.

### Adherence and biofilm formation assays

Adherence and biofilm formation abilities were assayed following a procedure previously described^52^. Briefly, overnight cultures containing either pJB3Tc19 and pJBpleD* or miniTn7pleD*Tc grown in LB medium supplemented with Tc were adjusted to an optical density (OD)_600_ of 0.04 into fresh LB medium before inoculation of 100 µL into 96-well microtiter plates and incubated for 2 h or 4 h at 28 °C statically. Loosely attached cells and medium were removed (for 2 h experiments, attached cells were fixed with 99% (v/v) methanol for 15 min and air-dried). Cells were stained with 100 µL of 0.1% (w/v) crystal violet solution for 20 min. The excess stain was removed by rinsing with distilled water. Crystal violet staining was quantified by eluting with 150 µL of 33% (v/v) acetic acid for 10 min and measured at OD_590_ in a Synergy™ HT Multi-Mode Microplate reader (BioTek Instruments). Experiments were repeated at least twice with 16 technical replicates.

### Competitive rhizosphere colonization assays

Alfalfa (*Medicago sativa* var. Resis) seeds were surface-sterilized with 70% (v/v) ethanol, 5% (v/v) sodium hypochlorite, washed gently with sterile distilled H_2_O, and germinated in H_2_O 1% (w/v) purified agar at 28 °C for 48 h. Seedlings were transferred and grown into 50 mL-tubes containing 25 mL of sterile pre-wetted medium-grain vermiculite and 10 mL Fåhraeus Plant (FP) medium^53^, in a controlled environment room (16/8 h, light/dark photoperiod and 25/18 ºC). Control and miniTn7pleD*Tc test strains were co-inoculated at 1·10^3^ colony forming units (CFUs) at a 1:1 ratio to one-week-old seedlings. *Pseudomonas ogarae* F113 wild-type and the *amrZ* mutant were used as controls. After a week post-inoculation, shoots were removed, and rhizosphere bacteria were recovered by resuspension of soil in 20 mL of NaCl 0.75% (w/v) by vortex. Dilution series were plated onto SA agar plates supplemented with Rif and Chx and allowed to grow for 72 h at 28 °C. CFUs of each strain were plated onto fresh SA plates and distinguished by resistance to antibiotics (Km in the case of the *amrZ* mutant and KmTc in the *amrZ* mutant with miniTn7pleD*Tc integration). Assays were performed in duplicate with ten independent plants each time.

### RNA isolation, cDNA synthesis, and qRT-PCR assay

Two independent cultures of F113 carrying the empty plasmid pJB3Tc19, *amrZ*^*-*^, *amrZ*^*-*^*fleQ*^*-*^ containing pJB3Tc19 or pJBpleD* were grown in SA medium until stationary phase. Then, 1 mL of each culture was centrifuged for 5 min at 12,000 × *g* at RT. The supernatant was discarded and the cell pellet was resuspended in 100 µL of RNAlater® Solution (Ambion) before storage at 4 °C. RNA isolation, cDNA synthesis, and qRT-PCR assays were custom made by Plataforma de Genómica de la Fundación Parque Científico de Madrid (Madrid, Spain) with the primers listed in Supplementary Table S3. Gene expression was calculated using the Ct values. Data were normalized using *rpoZ* expression as housekeeping and relativized to F113 pJB3Tc19 following the 2^−ΔΔCt^ method^54^.

### RNA-Seq

RNA isolation, sequencing, and bioinformatic analysis from F113 and *amrZ*^-^ cultures grown in SA medium at exponential (OD_600_ = 0.6) and stationary phase (OD_600_ = 1.2), or from rhizosphere colonization experiments were performed as described in^32^. F113 genes predicted to encode putative extracellular matrix components were selected. Differential gene expression was considered for values meeting a threshold of log_2_ Fold-Change (mutant/wild-type) ≥ 1 or ≤ -1 and a p-adjusted value ≤ 0.001. Heat-map was made using *pheatmap* R package version 1.0.12^55^.

### Statistical analysis

GraphPad Prism version 7.00 for Windows (GraphPad Software, San Diego, California USA, www.graphpad.com) was used in the statistical analysis and representation of swimming motility, biofilm formation assays, and qRT-PCR data using multiple t-tests for independent samples with Bonferroni-Dunn method.

## Supplementary Information

Supplementary Information.

## Data Availability

The transcriptome sequences of *Pseudomonas ogarae* F113 and derivatives used in this study are available at the National Center for Biotechnology Information (NCBI) under the BioProject accession number PRJNA419480: *P. ogarae* F113 in exponential (SAMN17839758 and SAMN17839763) and stationary (SAMN17839759 and SAMN17839764) growth phases and under rhizosphere conditions (SAMN17839757 and SAMN17839762); *amrZ* mutant in exponential (SAMN26939580 and SAMN26939582) and stationary growth phases (SAMN26939581 and SAMN26939583), and under rhizosphere conditions (SAMN17839760 and SAMN17839765).
