# Supplementary material for: Regulation of extracellular matrix components by AmrZ is mediated by c-di-GMP in Pseudomonas ogarae F113
Source: Sci Rep. 2022 Jul 13;12:11914. doi: 10.1038/s41598-022-16162-x (PMC9279365; doi:10.1038/s41598-022-16162-x)

# Regulation of extracellular matrix components by AmrZ is mediated by c-di-GMP in *Pseudomonas ogarae* F113

Esther Blanco-Romero, Daniel Garrido-Sanz, David Durán, Rafael Rivilla, Miguel Redondo-Nieto and Marta Martín

**Supplementary Table S1. Differential expression values of extracellular matrix-related genes in an *amrZ* mutant compared to *P. ogarae* F113 wild-type strain in different growth conditions** (Exp, Exponential; St, Stationary; Rhiz, Rhizosphere). $Log_2FC$ (Fold Change) and p-adjusted value are provided for each gene in every condition.

| Gene locus | Exp | | St | | Rhiz | |
|---|---|---|---|---|---|---|
| | $log_2FC$ | p-adjusted value | $log_2FC$ | p-adjusted value | $log_2FC$ | p-adjusted value |
| **PSF113_0161** | -4.89 | 2.54E-73 | -5.57 | 0.00E+00 | -2.40 | 5.12E-20 |
| **PSF113_0162** | -4.72 | 5.68E-99 | -5.55 | 0.00E+00 | -1.40 | 3.81E-06 |
| **PSF113_0163** | -4.85 | 1.53E-92 | -4.87 | 8.26E-161 | -0.86 | 1.11E-02 |
| **PSF113_0164** | -4.98 | 7.90E-38 | -4.93 | 7.08E-37 | -0.20 | 7.53E-01 |
| **PSF113_0208** | -1.89 | 0.00E+00 | -2.02 | 0.00E+00 | -1.00 | 0 |
| **PSF113_0209** | -4.72 | 1.98E-200 | -2.90 | 0.00E+00 | -1.05 | 4.94E-09 |
| **PSF113_0210** | -1.49 | 2.07E-46 | 1.87 | 4.86E-155 | -1.11 | 3.47E-85 |
| **PSF113_0211** | -0.95 | 3.94E-23 | 0.04 | 7.96E-01 | -1.17 | 3.26E-80 |
| **PSF113_1508** | -2.51 | 3.69E-225 | -2.23 | 1.19E-177 | -0.38 | 1.28E-04 |
| **PSF113_1509** | -3.41 | 6.25E-302 | -2.49 | 8.00E-197 | -0.41 | 7.49E-03 |
| **PSF113_1510** | -3.27 | 0.00E+00 | -2.56 | 0.00E+00 | -0.34 | 4.41E-03 |
| **PSF113_1511** | -4.61 | 0.00E+00 | -4.58 | 0.00E+00 | -1.24 | 1.19E-166 |
| **PSF113_1955** | -1.84 | 1.04E-06 | -2.67 | 8.11E-06 | -0.27 | 7.43E-01 |
| **PSF113_1956** | -2.79 | 9.28E-18 | -2.53 | 2.66E-13 | -0.85 | 1.23E-02 |
| **PSF113_1957** | -2.03 | 1.53E-23 | -2.54 | 5.25E-35 | -1.30 | 1.50E-02 |
| **PSF113_1958** | -2.64 | 1.26E-07 | -2.57 | 1.04E-10 | -0.87 | 1.01E-01 |
| **PSF113_1959** | -2.26 | 1.55E-03 | -3.24 | 7.30E-05 | -0.60 | 1.92E-01 |
| **PSF113_1960** | -2.88 | 2.22E-08 | -2.77 | 3.32E-08 | -0.94 | 1.87E-01 |
| **PSF113_1961** | -1.96 | 9.39E-10 | -2.36 | 3.42E-10 | -0.59 | 1.90E-01 |
| **PSF113_1962** | -1.87 | 1.71E-09 | -2.70 | 1.67E-12 | -0.56 | 4.78E-02 |
| **PSF113_1963** | -3.19 | 2.75E-12 | -1.56 | 5.85E-04 | -1.17 | 1.37E-02 |
| **PSF113_1964** | -4.17 | 9.53E-42 | -3.23 | 1.06E-25 | -1.68 | 1.47E-05 |
| **PSF113_1965** | -4.69 | 1.79E-178 | -3.49 | 3.04E-132 | -2.00 | 1.37E-11 |
| **PSF113_1966** | -4.71 | 5.24E-206 | -4.60 | 6.49E-108 | -2.06 | 1.29E-07 |
| **PSF113_1967** | -4.76 | 1.64E-122 | -4.20 | 6.64E-73 | -1.45 | 2.42E-03 |
| **PSF113_1968** | -5.58 | 6.09E-48 | -3.32 | 5.16E-22 | -2.18 | 9.29E-04 |
| **PSF113_1969** | -6.43 | 8.42E-35 | -6.18 | 2.84E-28 | -2.08 | 4.03E-03 |
| **PSF113_1970** | -6.17 | 1.67E-120 | -6.33 | 1.11E-118 | -0.98 | 2.92E-02 |
| **PSF113_2680** | 0.27 | 1.07E-03 | -0.66 | 5.79E-10 | -0.91 | 1.42E-11 |
| **PSF113_2681** | 0.08 | 9.03E-01 | -4.72 | 1.10E-07 | -2.64 | 4.40E-19 |
| **PSF113_2682** | -0.65 | 4.02E-01 | -4.01 | 8.80E-09 | -2.70 | 2.89E-13 |
| **PSF113_2683** | -0.52 | 3.31E-03 | -3.10 | 6.18E-144 | -3.36 | 9.23E-176 |
| **PSF113_2684** | -3.51 | 5.97E-02 | -5.64 | 2.84E-04 | -4.68 | 1.65E-07 |
| **PSF113_2685** | -3.92 | 2.82E-02 | -5.81 | 6.56E-08 | -2.20 | 5.59E-05 |
| **PSF113_3004** | -4.98 | 0.00E+00 | -5.42 | 0.00E+00 | -2.10 | 3.09E-107 |
| **PSF113_3005** | -2.17 | 7.01E-07 | -3.23 | 8.83E-06 | -0.54 | 1.65E-02 |

## Supplementary Table S1. Cont

| Gene locus | Exp | | St | | Rhiz | |
|---|---|---|---|---|---|---|
| | $log_2FC$ | p-adjusted value | $log_2FC$ | p-adjusted value | $log_2FC$ | p-adjusted value |
| **PSF113_3006** | -1.47 | 2.71E-05 | -3.49 | 1.86E-04 | -0.87 | 1.87E-06 |
| **PSF113_3007** | -1.45 | 2.27E-07 | -0.49 | 3.05E-01 | -0.77 | 3.02E-11 |
| **PSF113_4178** | -3.31 | 1.49E-125 | -1.43 | 6.28E-16 | -1.21 | 3.83E-25 |
| **PSF113_4179** | -3.54 | 1.25E-36 | -2.07 | 1.99E-08 | -0.91 | 1.35E-05 |
| **PSF113_4180** | -3.12 | 4.60E-15 | -2.94 | 2.28E-03 | -0.49 | 2.99E-01 |
| **PSF113_4181** | 0.39 | 3.14E-01 | 0.24 | 5.60E-01 | -1.73 | 2.72E-25 |
| **PSF113_4182** | 0.66 | 2.23E-02 | -0.08 | 8.70E-01 | -1.98 | 4.77E-25 |
| **PSF113_4183** | -0.02 | 9.36E-01 | -0.37 | 1.33E-01 | -1.77 | 2.73E-35 |
| **PSF113_4184** | -0.75 | 4.34E-04 | -0.26 | 5.03E-01 | -2.19 | 2.48E-36 |
| **PSF113_4185** | 1.52 | 1.01E-01 | 0.66 | 5.01E-01 | -2.04 | 2.49E-10 |
| **PSF113_4186** | -2.53 | 1.83E-01 | 0.29 | 8.87E-01 | -2.90 | 5.32E-07 |
| **PSF113_4187** | -1.72 | 8.82E-06 | -1.84 | 2.56E-03 | -2.61 | 1.17E-32 |
| **PSF113_4188** | -1.71 | 2.69E-20 | -0.70 | 6.99E-03 | -2.02 | 7.81E-22 |
| **PSF113_4189** | -3.03 | 1.22E-39 | -3.01 | 1.33E-23 | -1.82 | 1.81E-31 |
| **PSF113_4190** | -2.49 | 4.03E-08 | -2.82 | 3.17E-05 | -2.39 | 1.82E-28 |
| **PSF113_4191** | -2.85 | 1.64E-01 | 0.00 | 1.00E+00 | -3.17 | 1.17E-04 |
| **PSF113_4192** | -2.97 | 1.03E-01 | -3.44 | 6.66E-02 | -3.15 | 2.99E-08 |
| **PSF113_4752** | 1.10 | 7.38E-72 | 0.86 | 1.54E-29 | 0.54 | 7.64E-14 |
| **PSF113_4753** | 1.00 | 3.21E-10 | 0.70 | 9.16E-03 | 0.34 | 6.69E-03 |
| **PSF113_4754** | 1.52 | 7.35E-35 | 1.31 | 2.14E-16 | 0.26 | 1.02E-01 |
| **PSF113_4755** | 1.55 | 4.06E-48 | 0.64 | 1.89E-04 | 0.42 | 7.51E-03 |
| **PSF113_4756** | 0.44 | 4.75E-14 | 0.47 | 2.55E-13 | 0.11 | 4.14E-01 |
| **PSF113_4757** | 0.50 | 1.74E-10 | 0.09 | 4.45E-01 | 0.12 | 5.03E-01 |
| **PSF113_4758** | 0.87 | 2.18E-20 | 0.93 | 4.50E-14 | 0.37 | 5.49E-02 |
| **PSF113_4759** | 0.79 | 5.81E-19 | 0.73 | 3.26E-09 | 0.31 | 1.29E-01 |
| **PSF113_4760** | 0.70 | 3.13E-13 | 0.96 | 1.22E-10 | 0.37 | 7.98E-02 |
| **PSF113_4761** | 0.94 | 7.87E-41 | -0.08 | 5.32E-01 | 1.08 | 8.24E-17 |
| **PSF113_4762** | 0.82 | 5.57E-48 | 0.59 | 2.94E-16 | 1.18 | 1.58E-15 |
| **PSF113_4763** | 0.41 | 1.70E-60 | 0.20 | 3.42E-13 | 1.38 | 6.47E-84 |
| **PSF113_4764** | 0.80 | 1.82E-04 | -0.02 | 9.45E-01 | -0.54 | 8.48E-04 |
| **PSF113_4765** | -0.07 | 4.18E-01 | -0.74 | 9.04E-28 | 0.06 | 6.47E-01 |
| **PSF113_5195** | 0.38 | 4.90E-02 | 0.31 | 8.54E-02 | -0.10 | 6.92E-01 |

## Supplementary Table S2. Bacterial strains and plasmids used in this work

| Strains | Description | Reference |
|---|---|---|
| ***P. ogarae*** | | |
| **F113** | *P. ogarae* F113 wild type, $Rif^R$ | 1 |
| ***amrZ*$^-$** | *P. ogarae* F113 *amrZ*$^-$, $Rif^R$, $Km^R$ | 2 |
| ***amrZ*$^-$ *fleQ*$^-$** | *P. ogare* F113 *amrZ*$^-$*fleQ*$^-$, $Rif^R$, $Km^R$, $Gm^R$ | This work |
| **F113 - pJB3tc19** | *P. ogarae* F113 wild type, $Rif^R$, $Tc^R$, carrying pJB3tc19 | This work |
| ***amrZ*$^-$ - pJB3tc19** | *P. ogarae* F113 *amrZ*$^-$, $Rif^R$, $Km^R$, $Tc^R$, carrying pJB3tc19 | This work |
| ***amrZ*$^-$ *fleQ*$^-$- pJB3tc19** | *P. ogarae* F113 *amrZ*$^-$*fleQ*$^-$, $Rif^R$, $Km^R$, $Gm^R$, $Tc^R$, carrying pJB3tc19 | This work |
| **F113$^-$ - pJB*pleD**** | *P. ogarae* F113 wild type, $Rif^R$, $Tc^R$, carrying pJB3*pleD** | This work |
| ***amrZ*$^-$ - pJB*pleD**** | *P. ogarae* F113 *amrZ*$^-$, $Rif^R$, $Km^R$, $Tc^R$, carrying pJB3*pleD** | This work |
| ***amrZ*$^-$ *fleQ*$^-$- pJB*pleD**** | *P. ogarae* F113 *amrZ*$^-$*fleQ*$^-$, $Rif^R$, $Km^R$, $Gm^R$, $Tc^R$, carrying pJB3*pleD** | This work |
| **F113 Tn7*pleD**Tc** | *P. ogarae* F113, $Rif^R$, $Tc^R$, chromosomal integration of miniTn7*pleD**tc | This work |
| ***amrZ*$^-$ Tn7*pleD**Tc** | *P. ogarae* F113 *amrZ*$^-$, $Rif^R$, $Km^R$, $Tc^R$, chromosomal integration of miniTn7*pleD**tc | This work |
| ***E. coli*** | | |
| **DH5α** | *E. coli* cloning strain, φ80*lacZ*ΔM15, Δ(*lacZYA-argF*), U169, *recA1*, *endA1*, *hsd*R17 ($r_K^-$, $m_K^+$), *phoA*, *sup*E44-λ-*thi*-1, *gyrA96*, *relA1* | Gibco, BRL |
| **JM109** | *E. coli* cloning strain, *end*A1, *rec*A1, *gyr*A96, *thi, hsd*R17 ($r_k^-$, $m_k^+$), *rel*A1, *sup*E44, Δ( *lac-pro*AB), [F′ *tra*D36, *pro*AB, *laq*$I^q$ZΔM15] | Promega |
| **Plasmids** | | |
| **pJB3Tc19** | Cloning vector $Ap^R$ , $Tc^R$ | 3 |
| **pJB*pleD**** | pJB3Tc19 containing *pleD** from *Caulobacter crescentus* | 4 |
| **mini-Tn7*pleD**Tc** | pUC18T-mini-Tn7T with the EcoRI/SacI fragment containing *pleD**, $Ap^R$, and 1.3 Kb KpnI fragment containing Tc marker $Ap^R$ , $Tc^R$ | 5 |
| **pUX-BF13** | Helper plasmid providing the Tn7 transposition functions in trans, $Ap^R$ , $mob^+$, ori-R6K | 6 |
| **pRK600** | Helper plasmid, $Cm^R$ | 7 |

## References


1 Shanahan, P., O'Sullivan D, J., Simpson, P., Glennon, J. D. & O'Gara, F. Isolation of 2,4-diacetylphloroglucinol from a fluorescent pseudomonad and investigation of physiological parameters influencing its production. *Appl Environ Microbiol* **58**, 353-358, doi:10.1128/AEM.58.1.353-358.1992 (1992).

2 Martínez-Granero, F. *et al.* The Gac-Rsm and SadB signal transduction pathways converge on AlgU to downregulate motility in *Pseudomonas fluorescens*. *PLoS One* **7**, e31765, doi:10.1371/journal.pone.0031765 (2012).
3 Blatny, J. M., Brautaset, T., Winther-Larsen, H. C., Haugan, K. & Valla, S. Construction and use of a versatile set of broad-host-range cloning and expression vectors based on the RK2 replicon. *Applied and environmental microbiology* **63**, 370-379 (1997).
4 Pérez-Mendoza, D. *et al.* Responses to elevated c-di-GMP levels in mutualistic and pathogenic plant-interacting bacteria. *PLoS One* **9**, e91645, doi:10.1371/journal.pone.0091645 (2014).
5 Romero-Jiménez, L., Rodríguez-Carbonell, D., Gallegos, M. T., Sanjuán, J. & Pérez-Mendoza, D. Mini-Tn 7 vectors for stable expression of diguanylate cyclase PleD* in Gram-negative bacteria. *BMC microbiology* **15**, 1-10 (2015).
6 Bao, Y., Lies, D. P., Fu, H. & Roberts, G. P. An improved Tn7-based system for the single-copy insertion of cloned genes into chromosomes of gram-negative bacteria. *Gene* **109**, 167-168 (1991).
7 Finan, T. M., Kunkel, B., De Vos, G. F. & Signer, E. R. Second symbiotic megaplasmid in *Rhizobium meliloti* carrying exopolysaccharide and thiamine synthesis genes. *Journal of bacteriology* **167**, 66-72 (1986).

**Supplementary Table S3. Primers used in this work**

| Name | Sequence 5'-3' | Description |
|---|---|---|
| **rpoZ_F** | GTGGCAAAGAGCCGTTGGT | For qRT-PCR amplification of housekeeping gene *rpoZ* |
| **rpoZ_R** | TCAGCGTTGGCGATGAACT | |
| **pgaA_F** | TTCCTATCTGGAGCAGCGTGA | For qRT-PCR amplification of *pgaA* (PSF113_0161) from PNAG polysaccharide encoding operon (*pgaA-D;* PSF113_0161-0164) |
| **pgaA_R** | GCCTCTGCGGACTTTTCACTT | |
| **papA_F** | CCTCTACGGCTATCGCAAGGA | For qRT-PCR amplification of *papA* (PSF113_1970) from *papA-F* (PSF113_1970-1955) putative polysaccharide encoding cluster |
| **papA_R** | AAAATTCTTCCACTCGGTGCAG | |
| **mapA_F** | GCCTCGGTGGTGGAGGGCG | For qRT-PCR amplification of *mapA* (PSF113_1511), putative adhesin encoding gene |
| **mapA_R** | AAGTACGTCGTTGTCCAGC | |
| **psmE_F** | CGTGGTACCGGCCACCAG | For qRT-PCR amplification of *psmE* (PSF113_3004), putative adhesin encoding gene |
| **psmE_R** | GGGCGCTGCCGTTGGCGAG | |
| **lapA_F** | GTCGATAGCTCGGGCAGTATG | For qRT-PCR amplification of *lapA* (PSF113_208), adhesin encoding gene |
| **lapA_R** | CGACCAGGAAGATGTTTACGG | |
| **fapB_F** | GCGCTGTTTCTGCTCACTCTG | For qRT-PCR amplification of *fapB* (PSF113_2684) from Functional amyloids in *Pseudomonas* (Fap) encoding cluster (*fapA-F;* PSF113_2680-2685 ) |
| **fapB_R** | GCCTGGTTGACGTTGAAGTTG | |
| **flp1-F** | AAAGATGAAGACGGCCTGAC | For qRT-PCR amplification of the *low-molecular weight protein (flp)-1* gene (PSF113_4192). |
| **flp1-R** | TGCACGGATCTTGGTATTCA | |
| **alg8-F** | ACCTGATGGTCACCAGCTTC | For qRT-PCR amplification of *alg8* (PSF113_4753). |
| **alg8-R** | ATTTCCACGATGGAGCAGAC | |

**Supplementary Figure S1:** Biofilm formation on plastic tubes. Biofilm formation by *Pseudomonas ogarae* F113 and derivatives with (pJB3*pleD**) or without (pJB3tc19) c-di-GMP complementation. Strains were grown with shaking at 28 °C for 24 h in plastic tubes with SA medium supplemented with Tc (70 µg/mL). Crystal violet staining was performed directly in plastic tubes. Images were taken from the bottom of the tubes.

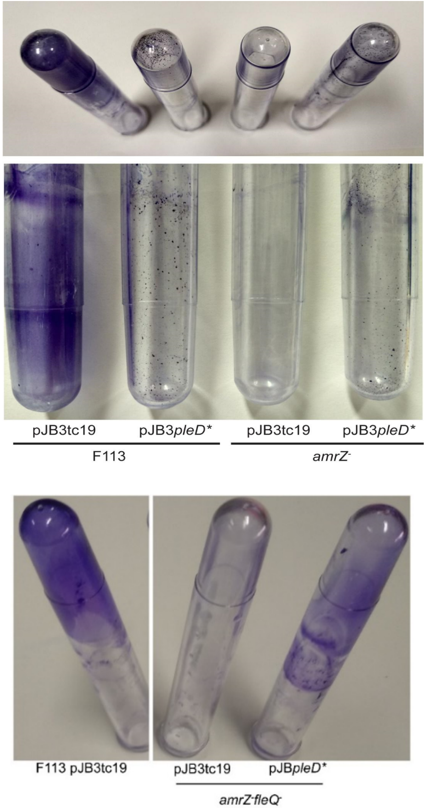


**Supplementary Figure S2:** Pellicles formation on plastic tubes. Pellicle formation by *Pseudomonas ogarae* F113 and derivatives with (pJB3*pleD**) or without (pJB3tc19) c-di-GMP complementation. Strains were grown under aerobic and static conditions at 28 °C for 72 h in plastic tubes with YMB medium supplemented with Tc (70 µg/mL).

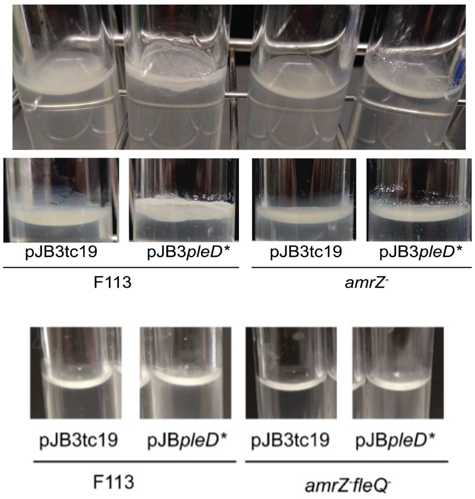

**Supplementary Figure S3:** C-di-GMP intracellular level complementation of biofilm and motility phenotypes in an *amrZ* mutant by MiniTn7*pleD**Tc chromosome integration. **(a)** Relative crystal violet-based biofilm formation assay of *P. ogarae* F113 and *amrZ*⁻ with or without miniTn7*pleD**Tc integration after 2 h (adherence) in microtiter plates with LB medium. The average and SD of two biological replicates with 16 replicates are represented; data was relativized to F113. Data were statistically analyzed with t-tests and Bonferroni-Dunn correction (significant differences: p-value<0.05). **(b)** Swimming motility assay of *P. ogarae* F113 and *amrZ*⁻ with or without miniTn7*pleD**Tc integration in SA medium. Average and SD of two biological replicates with three technical replicates are shown. Data were statistically analyzed with t-tests and Bonferroni-Dunn correction (significant differences: p-value<0.05).

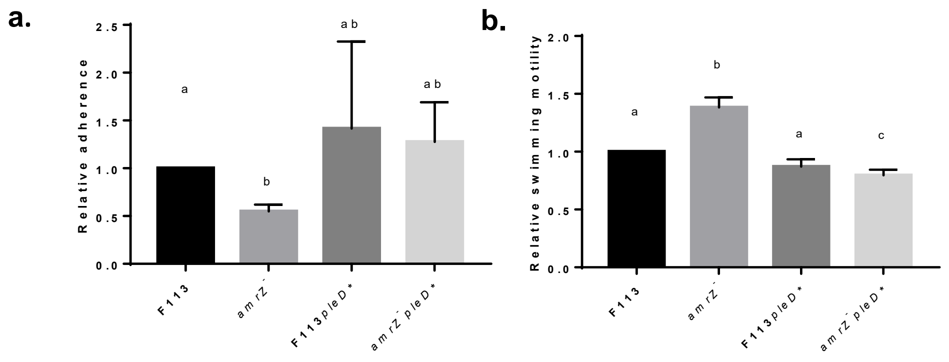

Supplement: Supplementary file 1 — Supplementary Information. [file 41598_2022_16162_MOESM1_ESM.pdf]
